# A Unified Global Reference Frame of Vertical Crustal Movements by Satellite Laser Ranging

**DOI:** 10.3390/s16020225

**Published:** 2016-02-08

**Authors:** Xinhui Zhu, Ren Wang, Fuping Sun, Jinling Wang

**Affiliations:** 1 Zhengzhou Institute of Surveying and Mapping, 66 Longhai Road, Zhengzhou 450052, China; chxy_wangren@163.com (R.W.); sun.fp@163.com (F.S.); 2 School of Civil and Environment Engineering, University of New South Wales, Sydney 2052, Australia; jinling.wang@unsw.edu.au

**Keywords:** vertical crustal movement, reference frame, SLR (satellite laser ranging), ITRF (International Terrestrial Reference Frame), systematic bias model

## Abstract

Crustal movement is one of the main factors influencing the change of the Earth system, especially in its vertical direction, which affects people’s daily life through the frequent occurrence of earthquakes, geological disasters, and so on. In order to get a better study and application of the vertical crustal movement, as well as its changes, the foundation and prerequisite areto devise and establish its reference frame; especially, a unified global reference frame is required. Since SLR (satellite laser ranging) is one of the most accurate space techniques for monitoring geocentric motion and can directly measure the ground station’s geocentric coordinates and velocities relative to the centre of the Earth’s mass, we proposed to take the vertical velocity of the SLR technique in the ITRF2008 framework as the reference frame of vertical crustal motion, which we defined as the SLR vertical reference frame (SVRF). The systematic bias between other velocity fields and the SVRF was resolved by using the GPS (Global Positioning System) and VLBI (very long baseline interferometry) velocity observations, and the unity of other velocity fields and SVRF was realized, as well. The results show that it is feasible and suitable to take the SVRF as a reference frame, which has both geophysical meanings and geodetic observations, so we recommend taking the SLR vertical velocity under ITRF2008 as the global reference frame of vertical crustal movement.

## 1. Introduction

Along with the high frequency occurrence of earthquakes and geological disasters, it is necessary to study crustal movements, especially in the vertical direction. Many experts have researched the vertical crustal movements in different areas and regions [1,2,3,4]. For instance, Brown and Oliver [1] studied the vertical crustal movements in the Eastern United States by levelling data of the Vertical Control Net carried out by the NGS (National Geodetic Survey). With the development of space geodetic techniques, people study vertical crustal movements and earthquakes through very long baseline interferometry(VLBI), SLR and GPS, sometimes integrating other geophysical methods [5,6,7,8]. Considerable effort and progress have also been made in the evolution of vertical crustal deformation models [9,10,11,12,13]. For example, Bevis and Brown [13] sketched the development of station trajectory models used in crustal movement and described some recent generalizations of these models, which gave us a clear understanding in geodesy and geophysics. However, the foundation and prerequisite for studying global vertical crustal movement is actually to establish its reference frame. Some scholars and data analysis centres have made some exploratory and confirmatory studies on the reference frame of vertical crustal motion.Mother *et al.* [14] pointed out that the geodetic reference system in four dimensions cannot be ignored because of the factors of Earth tides, plate motions, vertical crustal motions, and so on. Yurkina [15] indicated that some difficulties existed when establishing a reference system in vertical and crustal movement studies because of sea level changes considering variations of the Earth’s gravitational field and displacements of the Earth’s centre of mass (CM), and he also described the possibility by means of SLR techniques to study the vertical reference. Sun *et al.* [16] carried out a preliminary study of the reference frame of vertical crustal movements by using a postglacial rebound model. Dong *et al.* [17] indicated that an isostatic datum was inapplicable forstudying the present vertical crustal movement of the Chinese continent; therefore, a single-point dynamic vertical datum was discussed and established by using national high-precision levelling data. Zhang *et al.* [3,11,12] studied the datum of regional vertical crustal movement, which is based on the hypothesis of isostatic theory, and also analysed and fitted the deformation field of vertical crustal motion. As this method did not adopt global data, it was still a regional datum. Moreover, the results of global tectonic change proved that the hypothesis of an isostatic datum was not very accurate [2,18,19]. Jekeli and Dumrongchai [20] constructed the regional vertical datum IGLD85 (International Great Lakes Datum of 1985) by using satellite altimetry and level data. Bura *et al.* [21] realized a global vertical reference frame (GVRF) by using four regional and local vertical datums (LVDs) distributed worldwide through adopting the reference geopotential value, which was considered as a demonstration that the methodology proposed can be applied ata global scale. Amos and Featherstone [22] stated that New Zealand uses 13 separate local vertical datums based on geodetic levelling from 12 different tidegauges, and they attempted to use iterated quasi-geoid models to unify the vertical datum. Bevis *et al.* [23] presented a method for constructing and assessing the stability of a geometrical reference frame of vertical crustal movement. Donnelly *et al.* [24] and Haasdyk *et al.* [25] focused on the necessity and realization of a new geodetic datum for Australia and showed that if the geodetic datum is not updated, the systematic vertical bias will be 9 cm based on different datums. Crespi *et al.* [26] reviewed the definition and realization of geodetic reference frames and presented some relevant examples at both global and local levels, but did not expand the analysis of vertical reference frames, although which are widely applied. 

From the overall perspective of the Earth system, some definitions and conceptions, for example the GVRF and the global vertical datum (GVD) [27,28,29], have been put forward by several important organizations and symposiums, such as the IUGG (International Union of Geodesy and Geophysics), IAG (International Association of Geodesy), GGOS (Global Geodetic Observing System), IGGOS (Integrated GGOS), and so on. The above realizations are all complex with geometrically and geophysically-influenced factors and data collections, including the usual physical components (geoid and physical heights) and geometrical components (level ellipsoid and ellipsoidal heights). Vertical crustal movement is part of the change of the whole Earth system, so in this paper, we focus on the topic of the reference frame of vertical crustal movements, and we prefer to discuss it in ageometrical way and also include geophysical information based on the SLR technique itself.

A unified global reference frame of vertical crustal movements has a very significant meaning for a more rational and accurate global coordinate reference frame. Because of the disunity, the vertical velocity fields of global crustal movements are always distorted, even at the same co-located sites, and different techniques and data processing software applications give different results. Theoretically, vertical crustal movements should be defined as movements relative to the Earth’s CM. The vertical component of velocities in the same station should be the same. Actually, data processing based on different reference frames brings different results, as well. For the same station’s vertical component, different space geodetic data analysis centres around the world also publish different forms. For these reasons, the vertical data of various countries and regions in the world havebeen difficult to unify for a long time in the past, and such a situation will not only result in misunderstanding and confusion regarding application in the field of geodesy, but also restrict the observations used in various seismic, geological and geophysical fields and may even be misleading. Therefore, we definitely need to establish a unified global reference frame of vertical crustal movements in order to unify their velocity fields.

Velocity fields of global stations presented by different data analysis centres are almost constrained by the velocity vector based on the absolute plate motion model NNR-NUVEL1A (No net rotation-NUVEL1A) and established with one or more reference stations, while NNR-NUVEL1A can only give the station’s horizontal direction velocity [30,31], which indicates that its implicit constraint is that the vertical velocity of the reference station may be constrained aszero; for example, Watkins and Eanes [32] constrained the Kauai site’s vertical velocity to be zero according to the NNR-NUVEL1A model, which would have yielded vertical velocity uncertainties greater than 3 mm/yr. The actual situation is as follows: (1) the movement in the vertical direction of those reference stations changes by varying degrees; (2) the reference stations selected by different data analysis and processing centres are not exactly the same. The above conditions have caused disunity and the inaccurate use of the global vertical crustal movement reference frame. 

From the theoretical analysis, crustal vertical movements should be defined as a motion relative to the Earth’s CM, viz.the centre-of-mass of the total Earth system, which is the solid Earth and its fluid envelope and usually referred to as the geocenter [33]. The vertical velocity of one station should be the same, even when it is obtained by using different techniques, but in fact, due to the disunity of the reference frame of crustal vertical movements in different regions of the world, the results from some of the data analysis centres and regional data processing were different, and the resulting rate values differ even at the same station when they are obtained using different observation techniques, which limits the application of space observation techniques in national economical constructions. Therefore, it is necessary to unify the velocity fields of different data analysis centres and establish a uniform global reference frame of vertical crustal movements, which is currently an urgent problem in the field of global tectonics. This paper contributes by proposing a method of establishing the global reference frame of vertical crustal movements by using the SLR technique, which has a clear geophysical significance, is easy to implement and is suitable as a unified global reference frame of vertical crustal movements.

## 2. Method

### 2.1. Feasibility Analysis

After approximately 50 years of development, SLR is one of the most accurate space geodetic techniques: its single measurement precision has reached the sub-centimetre level, and it is currently the most accurate satellite tracking technique for monitoring the motion of the Earth’s CM [33,34]. SLR contributes to certain parameters of the reference frame, such as the origin and the scale [35,36], and the origin of the ITRF currently realized using only SLR data [33]. The ground station’s coordinates with respect to the Earth’s CM can be directly observed by the SLR technique; therefore, the station’s vertical velocity measured by SLR is also relative to the Earth’s CM. Since the movement of the Earth’s CM reflects the complexity of movement and interaction within the Earth's interior and various spheres, the vertical movement relative to the Earth’s CM can be seen as a reference frame of vertical crustal movement.

If the Earth’s CM is taken as a reference point, its motion should be considered as relative to the Earth’s centre. A geocenter refers to the CM of the entire Earth (including the oceans and atmosphere), which is unique. There are two motions of the Earth’s CM: one is the movement of the CM in space, such as the revolution of the Earth’s CM around the Sun; the other is in the Earth’s interior, which is caused by the change in the distribution of the Earth’s internal mass. The whole Earth (including the oceans and atmosphere) is a closed conservative system, because of the conservation of momentum, and thus, in theory, changes in the distribution of the Earth’s internal mass will not affect the motion of the Earth’s CM in space, but will only lead to variations of the geocentric position in the solid Earth’s interior. Currently, the hot research topic is intended to mean the latter geocentric movement.

Geocentric movement is a very complex process, including not only both short-term and short-period items, but also long-term or long-term periods. Sun *et al.* [37], based on the observations at the end of 19th centuryand theoretical calculations, indicated that at the time scale of 30 days to 10 years, the movement of the geocenter is within 1 cm; its primary cycle is annual, while its secondary one is six monthly, mainly due to the atmosphere, oceans and seasonal changes of surface water distribution. In accordance with the rule of movement of the Earth’s CM, an average geocentric position resolved in its primary cycle is called the conventional geocenter. A variety of short-term or periodic-term changes of the instantaneous geocenter relative to the conventional geocenter could be corrected by solving the stations’ mean coordinates, while long-term or long-cycle changes are reflected by the change of the station’s mean coordinates. Guo *et al.* [38] observed the distance of satellite LAGEOS 1/2 (LAserGEOdynamics Satellite) by using SLR, calculated the geocentric motion in the period 1993–2006, carried out an analysis and also found that a long and periodic change in the geocentric motion exists, where seasonal variation is the primary item, mainly through the mass distribution of the Earth fluid sphere, such as the oceans, atmosphere, land water, and so on. Thus, the change of geocentric movement is mainly reflected in a short-term or short-term period, while the long-term or long-term period did not change significantly, moreover when resolved, which can be reflected in the change of the station’s mean coordinates. In summary, when studying the station’s change in thelongterm or a long period, the geocentric centre can be used as a reference point; namely, it is feasible to take the SLR vertical movement relative to the centre of the Earth as a reference frame of vertical crustal motion.

ITRF2008 was released in 2010. The time span of observations adopted with the SLR technique is 1983.0–2009.0, and the observation epoch isat 2005.0. The error of the observation stations’ geocentric coordinate velocity is mostly within 1 mm/yr [6]. The velocity field obtained by the SLR technique under ITRF2008 is still based on time series, while compared toITRF2005, its observation accuracy and numbers have greatly improved. In summary, it is feasible to take the vertical velocity field obtained by the SLR technique under ITRF2008 as a global reference frame of vertical crustal movement. We discuss this in detail below and establish such a global unified reference frame of vertical crustal movements.

### 2.2. Establishment of Model

In theory, observation stations along the vertical direction of movement should be defined relative to the movement of the Earth’s CM, but in practical application, each data analysis centre has a certain arbitrariness in defining its vertical movement’s reference frame, mostly taking one or more stations’ vertical velocity located in a tectonically-stable region constrained to zero as the defining of the vertical reference frame, and therefore, the vertical movement after the reunification in the concept is still not relative to the centre of the Earth. The SLR technique can directly measure the geocentric coordinate and velocity field if a large number of SLR stations within high precisions are selected on a global scale, whose measured vertical velocities constitute the reference frame of the global vertical crustal movements. If the vertical velocity measured by SLR is relative to the Earth’s CM, a systematic bias between the other space-based observations and the SLR’s observations will inevitably exist. Therefore, in order to eliminate the systematic bias between the SLR measured velocity field and the other counterparts, it is possible to achieve a unified global reference frame of vertical crustal movements relative to the Earth’s CM. The systematic bias of different reference frames realized by different observed techniques is mainly reflected in the following aspects [39]:

(1) The coordinate origin is inconsistent. For example, the coordinate origin of VTRF (VLBI Terrestrial Reference Frame) is set by taking one SLR station’s geocentric coordinate defined relative to the Earth’s CM, and the station is co-located with VLBI and SLR, whose geocentric coordinate error will cause the deviation of the coordinate origin. In addition, different analysis centres may take different SLR stations to define the VTRF’s coordinate origin, which will lead to deviation of the origin between each VTRF and between the VTRF and other terrestrial reference frames. Although the terrestrial reference frames realized by SLR, LLR (lunar laser ranging), GPS and other space techniques are achieved by dynamic techniques, the dynamic reference systems adopted by various techniques are different, and therefore, the coordinate origin implemented by them may not be exactly the same. Besides, various dynamic techniques only determine the coordinate origin of the reference frame relative to the Earth’s CM to a certain precision.
(2) Scale is not entirely consistent. The scale of each terrestrial reference frame achieved by a variety of space techniques is jointly determined by the value of the speed of light C, the Earth’s gravitational constant GM(G is a gravitational constant, M represents the mass of the Earth) and the relativistic correction model. If each analysis centre uses different constants and models, this will lead to different scales for each terrestrial reference frame. Even if each analysis centre uses exactly the same constant and model, different observation techniques, different amounts of data and data processing methods also lead to a slightly different scale.
(3) Orientation differs. The defining axis point of the terrestrial reference frame is inconsistent when using different epochs and the Earth’s rotation parameters of different systems;there is a slight rotation between them. For example, although the BTS (Bureau International de l'Heure (BIH) Terrestrial System) andITRF sequence used BIH orientation parameters at epoch 1984.0, the other reference frame has adopted each defined parameter.

For two different frames, there are many transformation models when converting to the same frame. Here, we adopt the widely-used seven-parameter transformation and Hermelt transformation [23,40,41,42,43]. The transformation between two frames is as follows:

(1) $$X_{2}=X_{1}+T+{\text{DX}}_{1}+{\text{RX}}_{1}$$

where T (T_1_, T_2_, T_3_) are three translation parameters representing the difference of origins; R (R_1_, R_2_, R_3_) are three rotation parameters denoting the difference in orientation; D is the scale deviation. X_1_ and X_2_ are two vectors representing two different coordinates of one co-located site based on different reference frames obtained by two different observation techniques.

Due to the effect of plate motion, regional crustal deformation, postglacial rebound, and so on, station coordinates of the terrestrial reference frame will change over time. In order to maintain the stability of the conventional terrestrial reference frame, a high-precision reference frame always corresponds to a time epoch and a velocity field. The velocity field can be determined by a certain plate motion model (e.g., NNR-NUVEL1A [30,31] or APKIM2005 (ActualPlate Kinematic and crustal deformation Model) [44]) and can also be derived by observation results; nowadays a combination of the two is preferred. When the epoch of the conventional terrestrial reference frame changes, the transformation parameters will change, as well. Suppose that the rate of change of transformation parameters between two reference frames is $\dot{T}\left({\dot{T}}_{1},{\dot{T}}_{2},\dot{T_{3}}\right)$, $\dot{R}\left({\dot{R}}_{1},{\dot{R}}_{2},\dot{R_{3}}\right)$ and $\dot{D}$. Then, Equation (1) is derived by time *t*, and Equation (2) is obtained as follows:

(2) $${\dot{X}}_{2}={\dot{X}}_{1}+\dot{T}+\dot{D}X_{1}+D{\dot{X}}_{1}+\dot{R}X_{1}+R\dot{X_{1}}$$

Because the rotation matrix is typically less than 10 milliseconds, when transformed to radians, its magnitude is 10^−7^–10^−8^, and the order of the velocity field $\dot{X_{1}}$ is just a few centimetres per year, so the item $\dot{RX_{1}}$ in Equation (2) is relatively small and can be neglected. The scale parameter *D* is generally in the magnitude of 10^−6^–10^−7^, so it can be neglected, as well. Thus, Equation (2) can be simplified as follows:

(3) $${\dot{X}}_{2}={\dot{X}}_{1}+\dot{T}+\dot{D}X_{1}+\dot{R}X_{1}$$

The expanded Equation (3) is:

(4) $${\left[\begin{array}{c} \dot{X}\\ \dot{Y}\\ \dot{Z} \end{array}\right]}_{2}={\left[\begin{array}{c} \dot{X}\\ \dot{Y}\\ \dot{Z} \end{array}\right]}_{1}+\left[\begin{array}{c} \dot{T_{1}}\\ \dot{T_{2}}\\ \dot{T_{3}} \end{array}\right]+\dot{D}{\left[\begin{array}{c} X\\ Y\\ Z \end{array}\right]}_{1}+\left[\begin{array}{ccc} 0 & -\dot{R_{3}} & \dot{\;R_{2}}\\ \dot{R_{3}} & 0 & -\dot{R_{1}}\\ -\dot{\;R_{2}} & \dot{R_{1}} & 0 \end{array}\right]{\left[\begin{array}{c} X\\ Y\\ Z \end{array}\right]}_{1}$$

where ${\dot{X}}_{1}$ and ${\dot{X}}_{2}$ represent two velocity fields based on different reference frames, respectively.

The vertical movement surveyed by SLR is relative to the Earth’s CM. This means that there is a systematic bias between other velocity fields and the SLR velocity field. In order to obtain the systematic bias, co-located stations should be prepared using two techniques. Because different reference frames are adopted, the observation values of co-located stations are different. After systematic bias has been corrected, different velocity fields can be unified to a movement relative to the Earth’s CM. Systematic bias can be solved in an iterative manner, but the premise is to use the co-located data in two different reference frames. By eliminating the systematic bias in the geocentric reference frame and then minimizing the sum of the square of the projection of the difference between each vertical velocity and the SLR value on every co-located site in the geocentric direction, the systematic bias can be calculated by iteration using the following equation [19,45]:

(5) $$\overset{m}{\underset{i=1}{\sum }}{\left[\left({\vec{V}}_{i}^{1}-\vec{S}-{\vec{V}}_{i}^{2}\right)\cdot {\vec{X}}_{i}\right]}^{2}=min$$

where ${\vec{V}}_{i}^{1}$ and ${\vec{V}}_{i}^{2}$ are the projection vectors of the other technique’s vertical velocity field and the SLR’s one on the geocentric reference frame, respectively, $\vec{S}$ is the systematic bias, ${\vec{X}}_{i}$ is the unit geocentric vector of the *i*-th station and *m* is the number of stations.

If the velocity field is provided with site velocity in the geocentric reference frame, it can be transformed into a topocentric coordinate system along the directions of east, north and vertical using Equation (6):

(6) $$\left[\begin{array}{c} V_{i}^{e}\\ V_{i}^{n}\\ V_{i}^{u} \end{array}\right]=\left[\begin{array}{ccc} -\sin\lambda_{i} & \cos\lambda_{i} & 0\\ -\sin\varphi_{i} & -\sin\varphi_{i}\sin\lambda_{i} & \cos\varphi_{i}\\ \cos\varphi_{i}\cos\lambda_{i} & \cos\varphi_{i}\sin\lambda_{i} & \sin\varphi_{i} \end{array}\right]\left[\begin{array}{c} V_{i}^{x}\\ V_{i}^{y}\\ V_{i}^{z} \end{array}\right]$$

where $\varphi_{i}$ and $\lambda_{i}$ are the geocentric latitude and longitude of the *i*-th station, respectively, $V_{i}^{x}$, $V_{i}^{y}$ and $V_{i}^{z}$ are the three components of the geocentric velocity of the *i*-th station, respectively, and $V_{i}^{e}$, $V_{i}^{n}$ and $V_{i}^{u}$ are those of the topocentric velocity, respectively.

In Equation (5), the projection vectors of the velocity field’s vertical component on the geocentric reference frame are used in a systematic bias model. Therefore, vertical vectors should be calculated relative to the geocentric reference frame. The equation is as follows:

(7) $$\left[\begin{array}{c} v_{i}^{x}\\ v_{i}^{y}\\ v_{i}^{z} \end{array}\right]=\left[\begin{array}{c} \cos\varphi_{i}\cos\lambda_{i}V_{i}^{u}\\ \cos\varphi_{i}\sin\lambda_{i}V_{i}^{u}\\ \sin\varphi_{i}V_{i}^{u} \end{array}\right]$$

In Equation (4), $\dot{T}\left({\dot{T}}_{1},{\dot{T}}_{2},\dot{T_{3}}\right)$, $\dot{R}\left({\dot{R}}_{1},{\dot{R}}_{2},\dot{R_{3}}\right)$ and $\dot{D}$ denote the rate of change of systematic bias between two velocity fields based on different reference frames, while $\vec{S}$ in Equation (5) represents the systematic bias of two vertical velocity fields. Therefore, the systematic bias and the rate of change of two vertical velocity fields can be resolved from the combination of Equations (4) and (7).

If ${\dot{X}}_{1}$ represents the vertical velocity field obtained by the other technique, while ${\dot{X}}_{2}$ is that obtained by SLR, the two vertical velocity fields can be imputed to the geocentric reference frame by adopting Equations (4) and (7). Ignoring the impact of the rates of change of the rotation and translation parameters, the error equation can be given as follows:

(8) $$\left[\begin{array}{c} V_{1}\\ V_{2}\\ V_{3} \end{array}\right]=\left[\begin{array}{ccc} 1 & 0 & 0\\ 0 & 1 & 0\\ 0 & 0 & 1 \end{array}\begin{array}{ccc} 0 & -Z & Y\\ Z & 0 & -X\\ -Y & X & 0 \end{array}\begin{array}{c} X\\ Y\\ Z \end{array}\right]\left[\begin{array}{c} {\dot{T}}_{1}\\ {\dot{T}}_{2}\\ {\dot{T}}_{3}\\ {\dot{R}}_{1}\\ {\dot{R}}_{2}\\ {\dot{R}}_{3}\\ \dot{D} \end{array}\right]+\left[\begin{array}{c} L_{1}\\ L_{2}\\ L_{3} \end{array}\right]$$

where *X*, *Y* and *Z* are the Cartesian coordinates of the co-located station obtained by two different techniques and matrix *L* represents the difference between the projections of two vertical velocities in two different reference frames, as follows:

(9) $$\left[\begin{array}{c} L_{1}\\ L_{2}\\ L_{3} \end{array}\right]={\left[\begin{array}{c} \dot{X}\\ \dot{Y}\\ \dot{Z} \end{array}\right]}_{1}-{\left[\begin{array}{c} \dot{X}\\ \dot{Y}\\ \dot{Z} \end{array}\right]}_{2}$$

In addition, in order to analyse the difference between the SLR vertical reference frame and the other vertical velocity field, the fitting slopes of both results are calculated by the least squares linear fitting method using Equation (10), and the correlation coefficients are compared using Equation (11) [19,45]:

(10) y=a·x+b

(11) $$\rho_{xy}=\frac{cov\left(x,\;\;y\right)}{\sqrt{D\left(x\right)}\sqrt{D\left(y\right)}}$$

where *x* and *y* are the vertical velocities obtained by different space techniques, respectively, *a* and *b* are the fitting coefficientsand $\rho_{xy}$ is the correlation coefficient of the two results.

By using the fitting function and correlation, it is possible to analyse not only the difference between the vertical velocities obtained by SLR and the other technique, but also the consistency between velocity fields after correction by the systematic model, and then, by analysis and discussion, the reasonableness of taking the SLR vertical velocity as the reference frame of vertical crustal movement can be determined.

## 3. Data

The data used herein come from the ITRF, including the SLR, VLBI and GPS site coordinates and velocity fields in the ITRF2005 and ITRF2008 series, whose reference epochs are 2000.0 and 2005.0, respectively, solved by each data analysis centre. The following data sequence can be found on the official website of ITRF [46].

The SLR velocity field is chosen from the ITRF2008_SLR coordinate results submitted by each analysis centre. The solution deals with data from 1983.0–2009.0, comprising 26 years ofobservation data. The precision of station velocities is mostly better than 3 mm/yr.

The VLBI velocity field comes from ITRF2008_VLBI and ITRF2005_VLBI. The time sequence of ITRF2008_VLBI is from 1980.0–2009.0, comprising 29 years ofobservation data made up of the geocentric coordinates and velocities of VLBI stations. The reference epoch is 2005.0. The nominal precision of the geocentric coordinate is 3 mm. The precision of most geocentric coordinate velocities is better than 3 mm/yr. ITRF2005_VLBI.SCC adopted the 1992.9–2005 time series, a data span of nine years. The precision of most geocentric coordinate velocities is better than 3 mm/yr.

The GPS velocity field is from ITRF2008_GNSS and ITRF2005_GPS. The time span of ITRF2008_GNSS.SCC is from 1997.0–2009.5, comprising 12.5 years. The nominal accuracy of the geocentric coordinate obtained is better than 1 mm, and the station velocity accuracy is less than 1 mm/yr. ITRF2005_GPS.SCC contains GPS phase observation data from 1996.0–2006.0, and the nominal accuracy of most of its geocentric coordinates is better than 1 mm, while the accuracy of the stations’ horizontal velocities is mostly less than 0.2 mm/yr, and the vertical accuracy is better than 1 mm/yr.

Information about co-located sites was supplied by IERS (International Earth Rotation and reference systems Service) at 1 April 2010.

During data preprocessing, the following aspects are mainly considered: (1) whether the variance of station velocity of each component is better than 3 mm/yr; (2) whether the station is located in the border zone of the deformation plate or the internal plate’s apparent tectonic deformation zone; (3) whether the three components of the co-located site’s velocity remain in the same direction and of the same order of magnitude. By comprehensive application of the above criteria, the precision and stability of selected stations and co-located observations can be initially determined.

## 4. Resultsand Analysis

According to the above methods and observation data, the co-located sites (numbers of co-location sites shown in Table 5, Table 6, Table 7 and Table 8) of SLR, GPS and VLBI in different reference frames were selected, and then, the definition of the reference frame of global vertical crustal movements was studied by using the SLR vertical velocity in ITRF2008, which we defined as the SLR vertical reference frame (SVRF). The systematic bias between the SVRF and vertical velocities of GPS and VLBI, respectively obtained by using Equations (4), (5) and (7), namely the rate of change of transformation between them, is shown in Table 1, Table 2, Table 3 and Table 4.

In order to analyse the difference between SVRF and another vertical velocity, the method of linear regression analysis is adopted to resolve the regression coefficients between them. The fitting slopes and regression coefficients between the vertical velocities of SVRF, GPS and VLBI can be solved by using Equations (10) and (11), and the results are shown in Table 5, Table 6, Table 7 and Table 8, along with the fitting slope (*a*, *b*) and the regression coefficients between SVRF and the other velocity field before and after correction.

From the above Tables about the rates of change of transformation parameters, it can be seen that the systematic bias between the SVRF, VLBI and GPS velocities is very small, and the rate of change of transformation parameters is almost focused in the order of millimetres per year; the rotation parameters are milliarcseconds;and the scale parameter is smaller, as well: basically in the order of 10^−11^. Overall, the above obtained systematic biases are different in size and direction, which indicates that the reference frame of vertical crustal movement indeed does not unify the vertical velocities given by each data analysis centre. Comparing the systematic differences of Table 1 and Table 3, it can be shown that the reference datum of vertical crustal movements differs even with the same observation techniques at different observation epochs and time spans, and the same analysis results are obtained by comparing Table 2 to Table 4.

**Figure 1:**
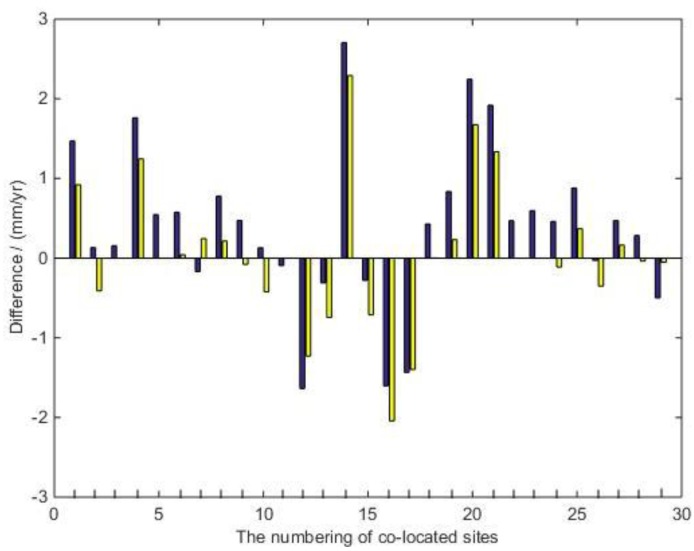
Differences between the ITRF2008_GNSS vertical-velocity field and the SLR vertical reference frame (SVRF) before (**blue**) and after (**yellow**) systematic bias correction.

From the above Tables about the fitting slope and correlation coefficient, the correlation coefficient has been improved after systematic bias correction; that is to say, the fitting degree between the SVRF and the vertical velocity of each technique after correction is better than before. In addition, we give an example to show the difference between them. Table 9 shows the comparison between SVRF and the velocity of ITRF2008_GNSS, as well as those corrections after systematic bias, respectively. Figure 1 shows the difference between the ITRF2008_GNSS vertical-velocity field and SVRF before and after systematic bias correction, where the left side is the difference between the SVRF and ITRF2008_GNSS velocity field, and the right is the difference after systematic bias correction in the corresponding representation. It can be seen from the above results that after the correction of systematic bias in the whole station, the consistency between SLR and the corresponding station’s vertical velocities is getting better. While the consistency of some stations isgetting worse, which indicates that, on the one hand, a difference between SLR and the other technique really exists, on the other hand, the process of solving systematic bias may also bring certain rounding errors.

The reference frame’s origin established by the SLR technique is defined relative to the Earth CM by taking the three first-order coefficients of the Earth’s gravitational field model as zero, so the observation of SLR is truly with respect to the Earth CM. From the basic principles of geodetic VLBI, VLBI is a pure geometric measurement, which is not sensitive to the Earth’s CM. In establishing the VLBI reference frame, the origin is typically determined through the co-location of the SLR station’s coordinates and relative baseline vector; that is to say, the reference frame-based VLBI technique is not truly relative to the Earth’s CM. Although high-precision GPS is a relative measurement, it is also solved by taking the Earth’s gravitational field’s three first-order coefficients as zero to ensure that the station coordinates are relative to the centre of the Earth. Therefore, in another sense, the reference frame established by the GPS technique is relative to the centre of the Earth, but it is not as direct as SLR; and the accuracy of the geocentric position obtained from GPS is not as good as SLR either [38]. Compared tothe SLR technique, the deficiencies in solar radiation pressure modelling of GNSS techniques affect the GNSS-derived geocenter series, especially the *Z* component [36,47]. Otherwise, the uncalibrated satellite antenna phase centre offsets of GNSS techniques are a major error source for the scale determination of the reference frame [36,48].

In summary, when taking the SLR vertical movement as the global reference frame of vertical crustal motion, the systematic bias between the SVRF and other velocity fields is relatively small. SLR is one of the most accurate space techniques for monitoring the geocentric motion, and it can directly measure the ground station’s geocentric coordinate and velocity relative to the centre of the Earth’s mass. Therefore, it is feasible to take the vertical velocity of the SLR technique obtained as a reference frame of vertical crustal motion for study, as it has the most direct geophysical significance and geometric meaning. Furthermore, with the progress of the SLR space observation technique, the precision and number of observations are constantly improving, and simultaneously, the station’s velocity field in the framework of ITRF2008 is based on the time series, so it is currently a reasonable and feasible implementation to take the SLR vertical velocity field as the unified global reference frame of vertical crustal movement.

## 5. Conclusions

Vertical crustal movement affects people’s living environment; therefore, it is of far-reaching importance to study and unify its reference frame on a global scale. This paper started from the feasibility of establishing a unified global vertical reference frame for crustal motion and then analysed and discussed the proposed method of taking the SLR vertical movement as the vertical reference frame, which was verified by the corresponding measured data. The conclusions are as follows:

The SLR technique is carried out by observing the distance from the ground station to the satellite to obtain the station’s location with respect to the centre of the Earth’s mass; namely, the ground station’s geocentric coordinate can be directly measured by the SLR technique, and thus, the station’s vertical velocity obtained by the SLR technique is relative to the centre of the Earth’s mass. Therefore, it is feasible to define the SLR vertical velocity field in the framework of ITRF2008 as the global reference frame of vertical crustal movement, which contains more extensive geophysical information, has a developed and improved characteristics, is directly relative to the centre of the Earth’s mass, and so on. In addition, the origin of the ITRF2008 framework is defined by using SLR data, so it is convenient and easier to unify the station’s coordinate frame and the vertical movement’s framework. Furthermore, Crespi *et al.* [26] underlined the unbreakable link between geodesy and geophysics; that is to say, geodesy requires the best models to explain geophysical phenomena, while geophysical models require continuous analysis of space geodetic observations. The SLR vertical reference frame is merged with both the geophysical meanings and geodetic observations, and thus, here, we recommend taking the SLR vertical velocity under ITRF2008 as the global reference frame of vertical crustal movement. In this paper, it is verified as the reference frame by using ITRF velocity field data. Because ITRF has already made internal integration of frames when implemented [6], the systematic bias solved here is smaller. In future work, we will use other forms of data to check whether the above method is reasonable or not, such as data from analysis centres on the world and solved in each of their frameworks. There is a referenced value, and the significance for the establishment of a unified global or regional reference frame of vertical crustal movements by the above systematically expounded related methods.

## Figures and Tables

**Table 1:** The rates of change of transformation parameters: ITRF2008_GNSS/SLR vertical reference frame (SVRF).

Translations (mm/yr)			Rotations (mas/yr)			Scale (10^−9^/yr)
${\dot{T}}_{1}$	${\dot{T}}_{2}$	${\dot{T}}_{3}$	${\dot{R}}_{1}$	${\dot{R}}_{2}$	${\dot{R}}_{3}$	$\dot{D}$
0.0473	−0.1017	0.1027	−0.0020	−0.0002	0.0004	0.0710

**Table 2:** The rates of change of transformation parameters: ITRF2008_VLBI/SVRF.

Translations (mm/yr)			Rotations (mas/yr)			Scale (10^−9^/yr)
${\dot{T}}_{1}$	${\dot{T}}_{2}$	${\dot{T}}_{3}$	${\dot{R}}_{1}$	${\dot{R}}_{2}$	${\dot{R}}_{3}$	$\dot{D}$
0.0065	−0.1758	0.1663	−0.0013	0.0006	0.0006	−0.0376

**Table 3:** The rates of change of transformation parameters: ITRF2005_GPS/SVRF.

Translations (mm/yr)			Rotations (mas/yr)			Scale (10^−9^/yr)
${\dot{T}}_{1}$	${\dot{T}}_{2}$	${\dot{T}}_{3}$	${\dot{R}}_{1}$	${\dot{R}}_{2}$	${\dot{R}}_{3}$	$\dot{D}$
0.2933	-0.1661	0.2628	−0.0030	−0.0039	0.0001	0.0339

**Table 4:** The rates of change of transformation parameters: ITRF2005_VLBI/SVRF.

Translations (mm/yr)			Rotations (mas/yr)			Scale (10^−9^/yr)
${\dot{T}}_{1}$	${\dot{T}}_{2}$	${\dot{T}}_{3}$	${\dot{R}}_{1}$	${\dot{R}}_{2}$	${\dot{R}}_{3}$	$\dot{D}$
1.5500	1.0856	0.8700	0.0198	−0.0299	0.0039	0.2845

**Table 5:** Fitting slope and correlation coefficient: SLR vertical reference frame (SVRF)/ITRF2008_GNSS.

Data Status	*a*	*b*	Correlation Coefficient	No. of Co-Location
Before Corrected	0.5740	0.5559	0.6404	29
After Corrected	0.5672	0.2095	0.7172	

**Table 6:** Fitting slope and correlation coefficient: SVRF/ITRF2008_VLBI.

Data Status	*a*	*b*	Correlation Coefficient	No. of Co-Location
Before Corrected	0.7062	−0.3142	0.7406	11
After Corrected	0.6953	−0.1381	0.7650	

**Table 7:** Fitting slope and correlation coefficient: SVRF/ITRF2005_GPS.

Data Status	*a*	*b*	Correlation Coefficient	No. of Co-Location
Before Corrected	0.9586	0.1143	0.8545	22
After Corrected	1.0117	0.3402	0.8602	

**Table 8:** Fitting slope and correlation coefficient: SVRF/ITRF2005_VLBI.

Data Status	*a*	*b*	Correlation Coefficient	No. of Co-Location
Before Corrected	0.8640	−0.2135	0.8976	9
After Corrected	1.2805	1.2598	0.9352	

**Table 9:** Velocity comparison: ITRF2008_GNSS/SLR vertical reference frame (SVRF).

Numbering of Sites	Site Name	Latitude (°)	Longitude (°)	Vu(mm/yr) before Corrected	Vu(mm/yr) after Corrected	Vu(mm/yr) SVRF
1	GRAS	43.7547	6.9205	0.9497	0.3984	−0.5234
2	GRAZ	47.0671	15.4934	0.9755	0.4328	0.8417
3	BOR1	52.2769	17.0734	−0.1985	−0.3555	−0.3553
4	CRAO	44.4132	33.9909	0.9494	0.4342	−0.8121
5	CAGL	39.1359	8.9727	0.6292	0.0828	0.0828
6	MATE	40.6491	16.7044	1.2091	0.6748	0.6317
7	HERS	50.8673	0.3362	−0.4892	−0.0754	−0.3211
8	SFER	36.4643	−6.2056	0.8938	0.3301	0.1145
9	ZIMM	46.8770	7.4652	1.8407	1.2892	1.3675
10	WAB2	46.9237	7.4642	1.4977	0.9459	1.3675
11	POTS	52.3792	13.0660	−0.3229	−0.2309	−0.2309
12	WTZR	49.1441	12.8789	−0.4793	−0.0707	1.1585
13	BJFS	39.6086	115.8924	2.6381	2.2052	2.9482
14	SHAO	31.0996	121.2004	1.2936	0.8806	−1.4123
15	KGNI	35.7103	139.4881	1.8650	1.4328	2.1445
16	MTKA	35.6795	139.5613	0.5400	0.1011	2.1445
17	HRAO	−25.8901	27.6869	−0.2437	−0.2067	1.1900
18	SUTH	−32.3802	20.8104	0.5283	0.1005	0.0988
19	ALGO	45.9558	−78.0713	4.1402	3.5374	3.3044
20	QUIN	39.9745	−120.9444	1.6741	1.1031	−0.5715
21	MDO1	30.6805	−104.0149	1.6503	1.0653	−0.2696
22	MAUI	20.7066	−156.2570	−0.6920	−1.1620	−1.1627
23	GODE	39.0217	−76.8268	−0.5481	−1.1447	−1.1446
24	MONP	32.8919	−116.4223	1.3620	0.7885	0.9013
25	AREQ	−16.4655	−71.4927	−0.9889	−1.5005	−1.8695
26	TIDB	−35.3992	148.9799	0.8747	0.5512	0.9016
27	YAR1	−29.0465	115.3469	0.3635	0.0571	−0.1080
28	STR2	−35.3161	149.0101	1.1871	0.8651	0.9016
29	THTI	−17.5770	−149.6064	−0.4598	−0.0133	0.0382

## References

[1] Brown L.D., Oliver J.E. (1976). Vertical crustal movements from leveling data and their relation to geologic structure in the Eastern United States. Rev. Geophys. Space Phys..

[2] Zhang Y. (1992). The discussion on isostatic instability condition of vertical crustal movement. Inland Earthq..

[3] Zhang Q., Zhao C. (2004). The spherical cap harmonic analysis method for crust vertical deformation field fitting. Acta. Geod. Cartogr. Sin..

[4] Koohzare A., Vanicek P., Santos M. (2008). Pattern of recent vertical crustal movements in Canada. J. Geodyn..

[5] Heki K. (1996). Horizontal and vertical crustal movements from three-dimensional very long baseline interferometry kinematic reference frame: Implication for the reversal timescale revision. J. Geophy. Res..

[6] Altamimi Z., Collilieux X., Metivier L. (2011). ITRF2008: An improved solution of the international terrestrial reference frame. J. Geod..

[7] Gu G., Wang W. (2011). Vertical crustal movement before and after the great Wenchuan earthquake obtained from GPS observations in the regional network. Earthquake.

[8] Hu Y., Qin S., Ming H. (2015). Three-dimensional crustal deformation before and after the Wenchuan earthquake in Guanzhong and adjacent regions. Geod. Geodyn..

[9] Holdahl S.H. Models for Extracting Vertical Crustal Movements from Levelling Data. http://adsabs.harvard.edu/abs/1978agtg.symp..183H.

[10] Liu Q., Chen Y. (1998). Combining the geodetic models of vertical crustal deformation. J. Geod..

[11] Zhang Q., Fan Y. (2001). The isostatic theory and the mathematical model of crust vertical movement. Acta. Geod. Cartogr. Sin..

[12] Zhang Q., Fan Y., Zhao C. (2004). Analysis model of crustal vertical movement based on the flux isostasy. Geomat. Inf. Sci. Wuhan Univ..

[13] Bevis M., Brown A. (2014). Trajectory models and reference frames for crustal motion geodesy. J. Geod..

[14] Mather R.S., Rizos C., Coleman R., Masters E.G. (1979). Geodetic reference systems for crustal motion studies. Tectonophysics.

[15] Yurkina M.I. (1993). Some reference problems in vertical crustal movement studies. J. Geodyn..

[16] Sun F., Li J. (1997). Definition and Realization of the Chinese Terrestrial Reference Frame. J. Inst. Surv. Mapp..

[17] Dong H., Wang W., Yao R. (2002). Establishment on the vertical datum of single point dynamic in China. Sci. Surv. Mapp..

[18] Sun F., Zhao M., Ning J., Chao D. (1999). Asymmetrical global tectonic changes based on the space geodetic measurements. Chin. Sci. Bull..

[19] Zhu X., Sun F. (2005). Detection of postglacial-rebound by using VLBI data. Chin. J. Geophys..

[20] Jekeli C., Dumrongchai P. (2003). On monitoring a vertical datum with satellite altimetry and water-level gauge data on large lakes. J. Geod..

[21] Bura M., Kenyon S., Kouba J., ŠÍMA Z., Vatrt V., Vojtíšková M. (2004). A global vertical reference frame based on four regional vertical datums. Stud. Geophys. Geod..

[22] Amos M.J., Featherstone W.E. (2009). Unification of New Zealand’s local vertical datums: Iterative gravimetric quasigeoid computations. J. Geod..

[23] Bevis M., Brown A., Kendrick E. (2013). Devising stable geometrical reference frames for use in geodetic studies of vertical crustal motion. J. Geod..

[24] Donnelly N., Dawson J., Evans G., Fraster R., Haasdyk J., Higgins M., Morgan L., Rizos C., Sarib R., Strong S., Turner S. Progress towards a New Geodetic Datum for Australia. Proceedings of the XXV International Federation of Surveyors (FIG) Congress.

[25] Haasdyk J., Davies L., Watson T. Progress towards a new geodetic datum for Australia. Proceedings of the 19th Association of Public Authority Surveyors Conference (APfAS2014).

[26] Crespi M., Mazzoni A., Colosimo G. (2015). Global and local reference frames. Rend. Fis. Acc. Lincei.

[27] Ihde J., Sánchez L. (2005). A unified global height reference system as a basis for IGGOS. J. Geodyn..

[28] Sjöberg L. (2011). On the definition and realization of a global vertical datum. J. Geod. Sci..

[29] Sánchez L. (2013). Towards a vertical datum standardization under the umbrella of Global Geodetic Observing System. J. Geod. Sci..

[30] Argus D.F., Gordon R.G. (1991). No-Net-Rotation Model of Current Plate Velocities Incorporate Motion Model NUVEL-1. Geophys. Res. Lett..

[31] DeMets C., Gordon R.G., Argus D.F., Stein S. (1994). Effect of recent revisions to the geomagnetic reversal time scale on estimates of current plate motions. Geophys. Res. Lett..

[32] Watkins M.M., Eanes R.J. (1994). Comparison of terrestrial reference frame velocities determined from SLR and VLBI. Geophys. Res. Lett..

[33] Wu X., Ray J., Dam T. (2012). Geocenter motion and its geodetic and geophysical implications. J. Geodyn..

[34] Yan F., Guo T., Wang P., Tan Y., Li X., Du R. (2006). Prospect of application of SLR on GALILEO plan and debris tracking. J. Geod. Geodyn..

[35] Wu X., Collilieus X., Altamimi Z., Vermeersen B.L.A., Gross R.S., Fukumori I. (2011). Accuracy of the international terrestrial reference frame origin and Earth expansion. Geophys. Res. Lett..

[36] Sośnica K., Thaller D., Dach R., Steigenberger P., Beutler G., Arnold D., Jäggi A. (2015). Satellite laser ranging to GPS and GLONASS. J. Geod..

[37] Sun F., Wu B., Yi W. Research progress in the motions of the Earth’s center of mass. Proceedings of the 7th National General Meeting of China Surveying and Mapping Academy.

[38] Guo J., Chang X., Han Y., Sun J. (2009). Periodic geocenter motion measured with SLR in 1993–2006. Acta. Geod. Cartogr. Sin..

[39] Sun F. (1994). Research of Current Crustal Motions Based on Space Geodetic Techniques. Ph.D. Thesis.

[40] Ray J.R., Ma C., Ryan J.W., Clark T.A., Eanes R.J., Watkins M.M., Schutz B.E., Tapley B.D. (1991). Comparison of VLB and SLR Geocentric Site Coordinates. Geophys. Res. Lett..

[41] Hofmann-Wellenhof B., Lichtenegger H., Collins J. (1994). Global Positioning System: Theory and Practice.

[42] Abusali P.A.M., Schutz B.E., Tapley B.D., Bevis M. (1995). Transformation between SLR/VIBL and WGS-84 reference frames. Bull. Géodésique.

[43] Boucher C., Altamimi Z. (2001). ITRS, PZ-90 and WGS 84: Current realizations and the related transformation parameters. J. Geod..

[44] Drewes H. (2009). The actual plate kinematic and crustal deformation model APKIM2005 as basis for a non-rotating ITRF Geodetic reference frames. Int. Assoc. Geod. Symp..

[45] Zhu X. (2005). Study on Space Geodynamics. Master’s Thesis.

[46] International Terrestrial Reference Frame. http://itrf.ensg.ign.fr/.

[47] Meindl M., Beutler G., Thaller D., Dach R., Jäggi A. (2013). Geocenter coordinates estimated from GNSS data as viewed by perturbation theory. Adv. Space. Res..

[48] Thaller D., Sośnica K., Dach R., Jäggi A., Beutler G., Mareyen M., Richter B. (2014). Geocenter coordinates from GNSS and combined GNSS-SLR solutions using satellite co-locations. Earth on the Edge: Science for a Sustainable Planet.

